# Metal Oxide Nanosensors Using Polymeric Membranes, Enzymes and Antibody Receptors as Ion and Molecular Recognition Elements

**DOI:** 10.3390/s140508605

**Published:** 2014-05-16

**Authors:** Magnus Willander, Kimleang Khun, Zafar Hussain Ibupoto

**Affiliations:** Department of Science and Technology, Campus Norrköping, Linköping University, Norrköping SE-60174, Sweden; E-Mails: kimleang.khun@liu.se (K.K.); zafar.hussain.ibupoto@liu.se (Z.H.I.)

**Keywords:** ZnO nanostructures, CuO nanostructures, NiO nanostructures, potentiometric nanosensors, biosensors, chemical sensors

## Abstract

The concept of recognition and biofunctionality has attracted increasing interest in the fields of chemistry and material sciences. Advances in the field of nanotechnology for the synthesis of desired metal oxide nanostructures have provided a solid platform for the integration of nanoelectronic devices. These nanoelectronics-based devices have the ability to recognize molecular species of living organisms, and they have created the possibility for advanced chemical sensing functionalities with low limits of detection in the nanomolar range. In this review, various metal oxides, such as ZnO-, CuO-, and NiO-based nanosensors, are described using different methods (receptors) of functionalization for molecular and ion recognition. These functionalized metal oxide surfaces with a specific receptor involve either a complex formation between the receptor and the analyte or an electrostatic interaction during the chemical sensing of analytes. Metal oxide nanostructures are considered revolutionary nanomaterials that have a specific surface for the immobilization of biomolecules with much needed orientation, good conformation and enhanced biological activity which further improve the sensing properties of nanosensors. Metal oxide nanostructures are associated with certain unique optical, electrical and molecular characteristics in addition to unique functionalities and surface charge features which shows attractive platforms for interfacing biorecognition elements with effective transducing properties for signal amplification. There is a great opportunity in the near future for metal oxide nanostructure-based miniaturization and the development of engineering sensor devices.

## 1. Introduction

Currently, the efforts of material researchers and engineers are primarily focused on the fabrication of cost effective and highly functional receptors that allow for a substrate to bind in a similar fashion as it is found in Nature. Selective recognition is the main component governing biological functions, such as immune reactions, and catabolic and anabolic enzyme-based reactions. In addition to high selectivity, natural systems have certain limits, including loss of activity in ambient conditions and reduced lifetime and stability. In the case of antibodies, which bind strongly to their respective antigens, the reversibility of the recognition is limited. However, the ionic interaction networks controlling biological reactions can be created to generate nanostructured artificial receptors with an extended lifetime. Different types of analyte-receptor bindings, including affinity-based binding, hydrophobic interactions, hydrogen bonding, and polar interactions, can be employed in the same manner. Especially for pure affinity interactions with the outer surface of the artificial receptor, the nanostructure-based recognition layer would strongly improve the sensor response because of the high surface-to-volume ratio of nanostructures [1–4].

Manmade materials have an affinity for protein recognition that is crucial for life sciences, particularly for proteomics studies and clinical examination. Biofunctional molecules, including receptors, antibodies, enzymes and different aptamers, have high capability for these applications; however, the limited resources prevent progress for a wide range of purposes. Recently, a considerable amount of work has been devoted to the synthesis of manmade materials for the functionalization of biomolecules. The synthesis of molecularly imprinted polymers is among these materials that are simple, cost effective and highly selective and have a strong attraction for target molecules [5–7]. Molecular imprinting is a well-known template-based polymerization technique to synthesize novel manmade materials with available recognition sites for target molecules. Molecular imprinted polymers for different small size molecules have been achieved by the aid of the co-polymerization of functional monomers and cross-linkers in the vicinity of template molecules [8–12]. More focus has been placed on the imprinting of proteins, which is a difficult task [13–15]. However, some materials possess excellent colors due to their hierarchically sequenced structures that are resistant to photo bleaching [16]. During the exposure to chemical vapors, these materials quickly change their colors because of changes in their structural and refractive indices [17]. Indeed, the materials that naturally exhibit colors and their synthetic derivatives are widely used because of their simplicity, and they provide a solid platform for the development of portable colorimetric sensors [18–23].

The detection of target molecules by the selective fluorescent technique in combination with a molecular imprinted polymer technique using a fluorescent reagent or quantum dots has been demonstrated to have excellent performance because of the high selectivity of molecular imprinted polymers and the sensitivity of fluorescence [24–27]. Moreover, the broadening of the nanotechnology field with potential applications for the molecular recognition of living organisms has created the foundation for wide-spread chemical sensing applications and the high throughput screening of ligand attachment. However, noticeable developments have been made using soluble proteins [28–31] and nucleic acids [32–34] combined with amphiphilic membrane proteins [35–37], irrespective of their importance and altered working performance in living organisms. G-protein coupled receptors consist of a large class of transmembrane receptors that quantitatively transport molecules into the intracellular environment and produce strong signal transduction pathways. These are actively involved in many diseases and are critical new drug targets. Olfactory receptor proteins are one class of G-protein coupled receptors whose transcripts account for approximately 3% of the mammalian genome [38]. In previous studies, olfactory receptor proteins were coupled with carbon nanotube transistors in which a direct chemical bond is formed that localize the olfactory receptors within nanometers of carbon nanotube-based devices. As a contrasting example, certain studies involve mobile membrane proteins that are introduced into a macroscopic lipid bilayer by coating the nanotube transistor. The results of these studies have enabled the direct readout of olfactory receptor ligand attachment by nanotube-based devices [39].

Additionally, molecular self-assembly techniques are very important for producing novel attractive materials with excellent features that are highly suitable for the design of sensor devices. These proposed materials are biomimetic at their core and have many similarities to their natural forms for the development of several hundred thousand nanostructures that are derived from all 20 amino acids [40]. For the regulation of cell functions, the patterning of chemical and topographic substrate surfaces is a vital technique. Lim *et al.* [41] described the comparative contribution of scale and patterns via chemical and topographical surfaces for the monitoring of cell functions. Chemical patterning can be based on spatial cell adhesive molecular organization. These patterns are capable of regulating various cell behaviors depending on their dimension of scale. However, topographic patterns either on a micro- or nano-scale control the specific cell reactions. Nano-dimension-based structures, including nanotubes, nanowires, nanorods, nanospheres, nanorings, nanoribbons, nanocomb, nanoflowers, nanofibers, nanoparticles, and nanocomposite materials, can be used to quantify biomolecules. The advantageous features of nanostructured materials include biocompatibility, lack of toxicity, large specific surface area, chemical and thermal stability, electro-catalytic activity and rapid electron communication, which are features of analytical tools with high sensitivity, selectivity, linearity, rapid response and reproducibility [42–45].

Among various sensor devices, potentiometric sensors are associated with attractive properties, such as ease of use, rapid response, low cost and immediate determination of the target analyte. Generally, potentiometric techniques are well known for the electrochemical transduction of ion selective sensor devices using a molecular imprinted polymer that functions as a selective molecular recognition membrane or as a layer in the sensor device. In this review, we discuss a few enzyme-, antibody- and membrane-based sensor devices using different metal oxide nanostructures as transduction elements for analyze recognition in our laboratory.

## 2. ZnO Nanostructure-Based Nanosensors

**Figure 1.**
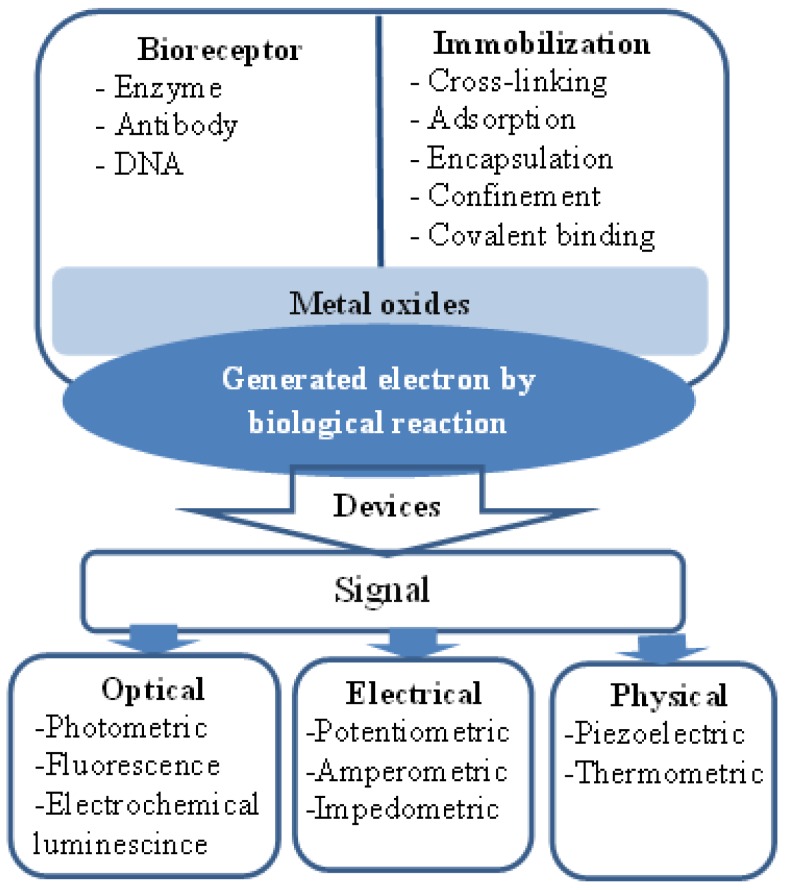
Schematic diagram for metal oxide nanostructure-based biosensing devices.

ZnO is a II-VI semiconductor material, and it exhibits various well known advantageous properties, such as biocompatibility, high specific surface area, chemical and photochemical stability, excellent light transmission, strong electrochemical and electron communication response and lack of toxicity. Hence, these properties have encouraged scientists to develop effective sensors. Moreover, ZnO has been given more preference over other metal oxide semiconductors due to the simplicity of the synthesis of various morphologies by different growth techniques [46,47]. Recently, the fabrication of electrochemical sensor devices using the favorable properties of biological and nanoscale-based materials has been considered a promising approach. The extraordinary high surface-to-volume ratio and surface activity of these devices have enabled nanostructures to be differentiated from the bulk material commonly used for enzyme immobilization and transducer usability. A sensor device is considered an analytical tool that has the ability to change a physical or chemical signal into an electrical or other signal using biosensitive material, such as enzymes, antibodies receptors, organelles and microorganisms, and the signal intensity, is associated with the concentration of the target analyte to be detected [48]. ZnO nanowires and nanorods are utilized effectively for the fabrication of chemical sensors, and both nanostructures have a high demand in environmental and industrial applications. Due to the high surface-to-volume ratio of nanowires and nanorods, these nanostructures have influenced a considerable change in the development of highly sensitive nanoscale-based chemical sensors [49–52]. The electrical, chemical and gas sensing characteristics of ZnO nanorods have been studied extensively, and it has been reported that the electrical transport properties of ZnO nanorods are strongly related to the adsorption/desorption properties of chemical substances [53–57]. Moreover, ZnO has a high isoelectric point of approximately 9.5, which provides a better microenvironment for the adsorption of low isoelectric point proteins because the immobilization of proteins is favored by electrostatic binding. It has been reported that positively charged ZnO nanorods have a tendency to enhance the direct electron transfer between the immobilized enzyme and the electrode at a significant rate [58]. Additionally, the high ionic property of ZnO makes it very useful for strong binding with the positively and negatively charged species. In addition to the use of ZnO nanostructures for sensing, they have also been used extensively for the fabrication of optoelectronics and optics because of their semiconducting, piezoelectric and piezoelectric features [59,60]. Moreover, the size of ZnO nanostructures is comparable to that of biochemical species, which makes ZnO an attractive primary transducer for the generation of strong electrical signals. The feasibility of using ZnO nanowires/nanorods has some limitations. For example, a chemical sensor based on a single nanorod is limited by the sensitive lithographic procedure, which requires considerable care during the fabrication of chemical sensors. The measureable change in current from a single nanorod requires costly instrumentation due to small changes in current during the adsorption/desorption phenomenon of the studied chemical substances. Moreover, single nanorod-based chemical sensors exhibit a significant difference in the measured current compared to similar analyzed samples because each nanorod has a different size (on the scale of nanometers) and also, the nature of electrical contacts affects the measured current value. These factors affect the difficult fabrication process for chemical sensors using a single nanorod [61]. However, due to the limitations described above, single nanorod-based chemical sensors have been recently designed using perpendicularly oriented nanorod arrays [62–64] wherein a metal contact is directly produced on the surface of nanorod arrays by a sputtering technique [61,63] or an aerosol spray pyrolysis method [63]. This approach of chemical sensor fabrication is also limited by the gradient interfaces between nanorods and metal electrodes; these interfaces diminish the effectiveness of sensor devices. Our group is actively addressing the fabrication of various morphologies of ZnO materials and their potential applications. Among the different morphologies, nanorods have been used in various products, such as white light emitting diodes, ultraviolet photodiodes, chemical sensors and biosensors [65–68]. For example, Ibupoto *et al.* [69] have developed a potentiometric L-lactic acid biosensor based on lactate oxidase immobilized ZnO nanorods for the selective determination of L-lactic acid. The proposed L-lactic acid biosensor exhibits a wide range of detection of lactic acid concentration (1 × 10^−4^ to 1 × 10^0^ mM), good sensitivity (41.33 ± 1.58 mV/decade) and a rapid response time of less than 10 s. A schematic diagram for the fabrication of electrochemical biosensors is shown in Figure 1.

## 3. Penicillinase Enzyme Immobilized ZnO Nanorods for the Selective Detection of Penicillin Using Potentiometric Technique

**Figure 2.**
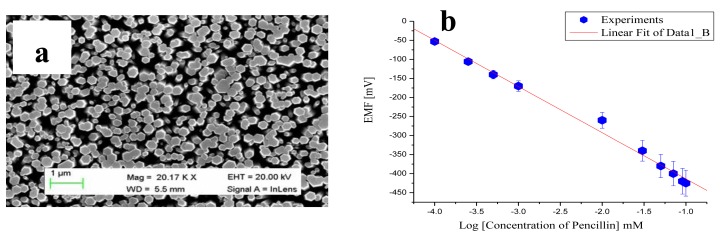
(**a**) SEM image of ZnO nanorods and (**b**) calibration curve of penicillin sensors [70].

After the successful synthesis of ZnO nanorods using a hydrothermal method, the nanorods were immobilized with the penicillinase enzyme for the selective determination of penicillin. An SEM image of ZnO nanorods is shown in Figure 2a. Six penicillin biosensor electrodes were prepared independently by immobilizing penicillinase enzyme on the ZnO nanorods using the electrostatic adsorption method of immobilization. The immobilization was conducted in two steps. First, a 10 mM solution of N-5-azido-2-nitrobenzoyloxysuccinimide (ANB-NOS) cross-linking molecule was prepared in a phosphate buffer solution of pH 7.4 and nanorods were subsequently dipped in this solution for approximately 1 h. Then, the nanorods were cleaned with deionized water and dried at room temperature. Next, the cross-linking molecule-bound ZnO nanorods were vertically maintained in the penicillinase enzyme solution containing 5 mg/mL penicillinase for 20 min. Afterwards, the enzyme-immobilized ZnO nanorods were kept at 4 °C for 16 h.

The potentiometric response of the proposed sensor electrode was measured at room temperature using penicillinase enzyme-immobilized ZnO nanorods as a working electrode against silver-silver chloride (Ag/AgCl) as a reference electrode. The output potential response produced between penicillinase-immobilized ZnO nanorods and Ag/AgCl is directly related to the specific concentration of penicillin inside the solution at the set temperature. The observed change in output potential also depends on the ability of the penicillinase enzyme to hydrolyze penicillin salt. The enzyme-based hydrolysis reaction of penicillin G salt can be described as follows:

(1) $$\text{Penicillin}+{\text{H}}_{2}\text{O}={\text{penicilloate}}^{-}+{\text{H}}^{1+}$$

**Figure 3.**
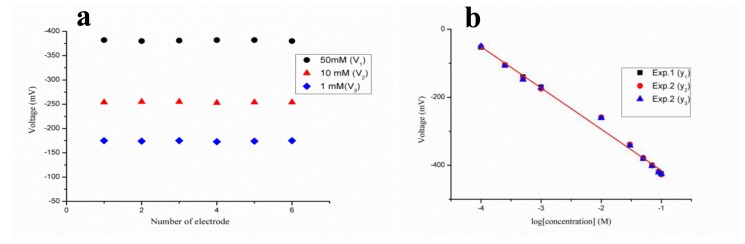
(**a**) Reproducibility of the penicillin sensor across three different concentrations of penicillin and (**b**) repeatability of the penicillin sensor using one electrode in triplicate [70].

The reaction above shows the product of two charged species—hydronium and penicilloate ions—during the enzymatic reaction, and the concentration of penicillin can be estimated by considering either the hydronium ion or penicilloate ion concentration. The output potential is related to the change in the concentration of charged species near the working electrode [71]. The potentiometric technique was used for the measurement of electrochemical response, and the output response of the penicillinase-immobilized ZnO nanorod-based electrode was measured for the 100 μM to 100 mM concentrations of penicillin G salt as shown in Figure 2b. The proposed penicillin sensor demonstrated the wide range of detection of penicillin with high selectivity and a sensitivity of 121 mV/decade. An acceptable sensor device has good reproducibility, linear range of detection, selectivity, repeatability and a rapid response time. The reproducibility describes the consistency of different sensor electrodes prepared under the same set of conditions. In the present case, six independent sensor electrodes were prepared by immobilizing the penicillinase enzyme on the surface of ZnO nanorods to monitor the reproducibility and stability of the penicillin sensor device throughout its lifetime. The potentiometric response of each penicillin sensor electrode was measured in 1, 10 and 50 mM concentrations of penicillin, and the sensor device exhibits excellent reproducibility with a relative standard deviation of less than 5% as shown in Figure 3a.

**Figure 4.**
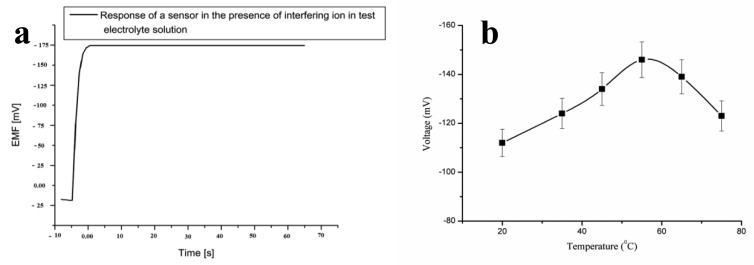
(**a**) Time response curve of the proposed sensor in a 1000 μM penicillin electrolyte test solution in the presence of interfering species; and (**b**) effect of temperature on the biosensor from 20 to 75 °C [70].

The present penicillin sensor based on penicillinase-immobilized ZnO nanorods has also been shown to have good repeatability after the use of the same sensor electrode for three days. The sensor electrode was washed with phosphate buffer solution prior to the experiment and after the experiment, and the sensor device was stored at 4 °C when it was not in use. The obtained result for the repeatability is shown in Figure 3b wherein it can be observed that the sensor has a good repeatable response for the detected concentration range. The selectivity of a particular biosensor describes its selective response in the presence of other substances under the same set of conditions. In the present case, the selectivity of the penicillin biosensor was examined by the change of response after the addition of common interfering species as shown in Figure 4a.

It has been reported that penicillinase is highly selective towards penicillin [72], and in the presence of penicillinase, the rate of the hydrolysis reaction of penicillin G salt is greatly increased, which results in the selective response for the determination of penicillin levels. The penicillin biosensor has exhibited a penicillin-specific response in the presence of common interferences. The proposed biosensor has shown a negligible response for Na^+1^, K^+1^, D-glucose, L-glucose, ascorbic acid, uric acid, urea, sucrose, lactose, glycine, penicilloic acid, and cephalosporins interferences. Figure 4a shows the response of a penicillin biosensor based on penicillinase-immobilized ZnO nanorods by adding a 100 μM concentration of each interfering substance in 1 mM penicillin G salt, and no effect on the output signal was observed. It can also be observed that the sensor electrode exhibited a rapid response time of less than 5 s. The lifetime stability of the proposed penicillin sensor electrode was investigated for more than four weeks by conducting a regular series of experiments. The sensor electrode was stored at 4 °C when not in use. The sensor electrode has the ability to maintain its function for a longer period of time.

The change in output signal with respect to temperature was also examined to demonstrate the maximum response of the present sensor electrode at a specific temperature. Therefore, a series of temperatures were selected from 20 °C to 80 °C as shown in Figure 4b. It can be inferred from Figure 4c that the sensor electrode has a maximum output signal at 50 °C. Afterwards, the response decreased. The increasing response of the sensor electrode could be due to the maximum activity of penicillinase enzyme at 50 °C, and the decreasing trend at temperatures above 50 °C could be due to the denaturation of protein molecules of penicillinase at high temperatures. Although the sensor electrode exhibited a maximum response at 50 °C, all the experiments were carried out at room temperature due to the ease in conducting the experiments and to avoid the possible evaporation of the electrolyte solution.

## 4. Antibody Immobilized Metal Oxide Nanostructures-Based Immunosensors

Immunosensors are gaining considerable attention for the quantification of proteins, biological toxins, biomarkers and biowarfare species in emergency situations, including food security, pharmaceutical chemistry, environmental monitoring and clinical applications. Immunosensors are typically developed utilizing antibodies or their complementary binding counterparts as biorecognition elements in addition to electrochemical transducers [73].

The highly charged surface of metal oxide nanostructures and effective electron transfer rate properties support these nano dimensions and facilitate the immobilization of antibodies. An interesting property of metal oxide nanostructures is that the Fc terminal of an antibody is attached to these morphologies, whereas the Fab terminal is unbound and can thus bind with the antigen with enhanced selectivity. For the fabrication of immunosensors based on metal oxide nanostructures, antibodies of interest are immobilized onto an electronically conducting electrode, including ITO. The sensing ability of a device is based on the density of antibodies immobilized on a more favorable surface. There is ample opportunity for the fabrication of immunosensors to be improved using new chemical and physical procedures to enhance the functionality, electrical conductivity and morphology of biocompatible metal oxide nanostructures [73,74]. A thin film of IrO_2_ synthesized electrochemically on a metal surface acts as a substrate for the development of immunosensor electrodes, and it has been used in immunoassays for immunoglobulin G (IgG) [75]. The iridium oxide thin film was hydrophilic and exhibited significant porosity with a high specific surface area. The stability of this immunosensor has not been explored yet. A graphite electrode consisting of single walled nanotubes and a self-assembled Nafion/Fe_2_O_3_ was used to immobilize anti-biotin antibodies for the determination of HRP-labeled and unlabeled biotin [76]. The fabricated immunosensor exhibited a low limit of detection because of the high specific surface area of single walled carbon nanotubes and the high conductivity of the electrode. However, the sensing performance of this device has not yet been studied. Hafaid *et al.* [77] developed an immunosensor using Fe_3_O_4_ nanoparticles for biotin-streptavidin interaction. The surface plasmon resonance technique was used to measure the deviation angle at the time of antigen-antibody recognition. Mantzila *et al.* fabricated an anodic Ti/TiO_2_ (anatase) electrode at specific potentials using an impedimetric technique [78]. Wang *et al.* [79] developed an immunosensor using low temperature aqueous synthesized TiO_2_ nanowires modified on a gold microelectrode through mask welding. The TiO_2_ nanowires were functionalized with monoclonal antibodies and the resulting device was found to be sensitive, specific and rapid in the determination of *Listeria* monocytogenes for concentrations at low levels of 102 cfu·mL^−1^ in 1 h without any detectable interference from other food-borne pathogens.

**Figure 5.**
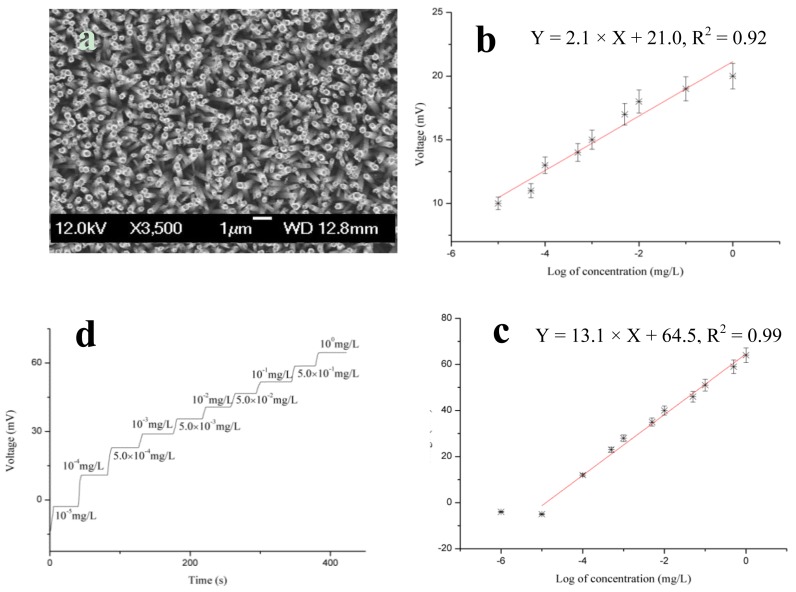
(**a**) SEM image of ZnO nanotubes; (**b**) calibration curve of bare ZnO nanotubes for CRP-antigen; (**c**) calibration curve of antibody-immobilized ZnO nanotubes for CRP-antigen; and (**d**) response time for all concentrations of the CRP-antigen biosensor [82].

TiO_2_ nanostructures have been used for the fabrication of label-free optical interferometric sensor devices by studying specific proteins [80]. The electrostatic interaction was analyzed for the immobilization on the negatively charged surface of TiO_2_ nanostructures and an IgG sensing element for trinitrotoluene quantification in the pH range of 2–12. The modified TiO_2_ substrates were immobilized with antibodies for prostate specific antigens [81]. After the successful synthesis of ZnO nanotubes through chemical etching of ZnO nanorods synthesized by a hydrothermal method, anti-C-reactive protein was physically adsorbed on the ZnO nanotubes for the selective detection of C-reactive protein [82]. A typical SEM image of ZnO nanotubes is shown in Figure 5a. The brief immobilization was followed by the dissolution of antibody in 1 mM phosphate buffer solution at a 15 × 10 mg/L antibody concentration. To prevent the self-antibody reaction, 2.5% glutaraldehyde was mixed with the antibody solution, and ZnO nanotubes were immersed in the antibody solution for 5 min. Afterward, the antibody immobilized ZnO nanotube-based electrodes were dried at room temperature for 1 h and stored at 4 °C overnight. The electromotive force of the fabricated sensor device was measured by a potentiometric technique. During the insertion of antibody immobilized ZnO nanotubes in the C-reactive protein solution, complex formation occurs between the immobilized antibody and C-reactive molecules, and an output potential was recorded by pH meter model 728. The cell assembly was based on a two electrode system; the working electrode consisted of the ZnO nanotubes functionalized with antibody and Ag/AgCl as the reference electrode. ZnO nanotubes were used as a primary transducer element for the immobilization of antibody and also to catalyze the complex formation between the immobilized antibody and C-reactive protein molecules. The electrostatic interaction between the ZnO nanotubes and protein molecules can be explained in terms of the high isoelectric point of ZnO, which was approximately 9.5, indicating the strong attraction for low isoelectric point molecules, such as antibodies, in the present case.

The experiment to evaluate the use of antibody-immobilized ZnO nanotubes for the selective detection of C-reactive protein was conducted using different concentrations of C-reactive protein. The potentiometric response was measured for the concentration range of 1.0 × 10^−6^ to 1.0 × 10^0^ mg/L C-reactive protein. Linear detection was observed for the concentration range of 1.0 × 10^−5^ to 1.0 × 10^0^ mg/L. The concentrations of C-reactive proteins were prepared in 1 mM phosphate buffer solution. To confirm the complex formation between the immobilized antibody and C-reactive protein, we used a sensor electrode of bare ZnO nanotubes and we added them to the testing solution of C-reactive protein. The response is shown in Figure 5b. This measurement showed that the bare ZnO nanotubes exhibited a negligible output potential response for the detection of C-reactive protein; however, a similar measurement was performed with the antibody-immobilized ZnO nanotubes using a similar concentration range of C-reactive protein. The fabricated device exhibited a rapid and stable electromotive response as shown in Figure 5c. This could be due to the rapid complex formation between the immobilized antibody and C-reactive protein. ZnO has an approximately 60% ionic character, which aids the stable binding of antibody molecules with the surface of ZnO nanotubes and further catalyzes the rapid complex formation between the immobilized antibody and C-reactive protein molecules. Figure 5c represents the calibration curve for C-reactive protein molecules. The curve demonstrates good linearity with a sensitivity of 13.17 ± 0.42 mV/decade. The antibody-immobilized ZnO nanotube-based sensor device was also examined in a solution of a non-specific protein, bovine serum albumin (BSA), instead of C-reactive protein to analyze the negative role of the currently described immunosensor device. The results of this study showed that the sensor device has the ability to only selectively respond to the C-reactive molecules, and the results also revealed a negligible response for non-specific proteins, such as BSA. The antibody-immobilized ZnO nanotube-based immunosensor exhibited a rapid response time of less than 10 s when used in the entire detected range of C-reactive protein molecules as shown in Figure 5d.

**Figure 6.**
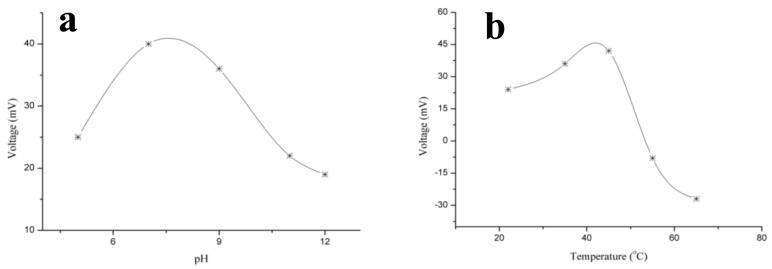
(**a**) Effect of pH on the output response of the CRP-antigen biosensor and (**b**) effect of temperature on the output response of the CRP-antigen biosensor for the temperature range of 25 °C to 75 °C [82].

Figure 6a shows the effect of pH on the output potential of a fabricated sensor device when the pH of the testing solution was altered. The pH of a C-reactive protein solution at a concentration of 1 × 10^−2^ mg/L was adjusted by adding 0.1 M hydrochloric acid (HCl) and 0.1 M sodium hydroxide (NaOH). It can be observed that the maximum output potential was obtained near pH 7, which is very close to 7.2, the optimum pH for antibodies. However, for higher pH values, the currently described sensor device exhibited a low output potential response, which could be attributed to the decreased activity of the antibody used for this study. A low output potential response was obtained for pH below 7, which could be attributed to the loss of antibody molecules resulting from the instability of ZnO nanomaterial at low pH values.

In addition to the pH effect, temperature also has a significant influence on the output potential of surface-based immunosensors because of the variation in the mobility of charge on the surface of the transducer. Figure 6b shows the effect of temperature on the electromotive response of the currently described sensor device for the temperature range of 25 °C to 75 °C, and the maximum output signal was observed at 55 °C. At temperatures above 55 °C, low output potential was observed due to the denaturation of the functionalized antibody on the surface of ZnO nanotubes. However, all the measurements were performed at room temperature because of the controlled nature of the experimental setup and also to prevent the evaporation of the testing solution. The performance evaluation of a particular sensor device is examined by certain parameters, such as repeatability, reproducibility, lower limit detection, selectivity, stability and sensing time for the target analyte. The repeatable response of the fabricated sensor device is identical to the response of the sensor electrode that was used more than once under a similar set of experimental conditions. The immunosensor discussed here exhibits good control over the detection range of analytes with similar sensitivity. The sensor device was stored at 4 °C when not in use. The immunosensor was reproducible for different concentrations of C-reactive protein. The immunosensor is highly reproducible, and a relative standard deviation of less than 5% was observed. Selectivity is the main parameter used to evaluate the performance of the sensor device because it indicates the sensing ability of the sensor device for the target analyte. The separation solution method was followed for the measurement of the selective response of the presented sensor device [83].

The interference response was measured for the common interferences present in human serum, and we observed a negligible response towards those interferences. The storage lifetime of a particular sensor device depends on the conditions during the measurement, and during this investigation, we determined that a sensor device could not be used for more than three days; thus, we concluded it can function as a disposable device. The metal oxide nanostructures are highly valuable for the fabrication of immunosensors by immobilizing the monoclonal antibodies for the selective detection of antigens.

## 5. Polymeric Membrane Coated ZnO Nanorod-Based Thallium (I) Ion Sensing Application

**Figure 7.**
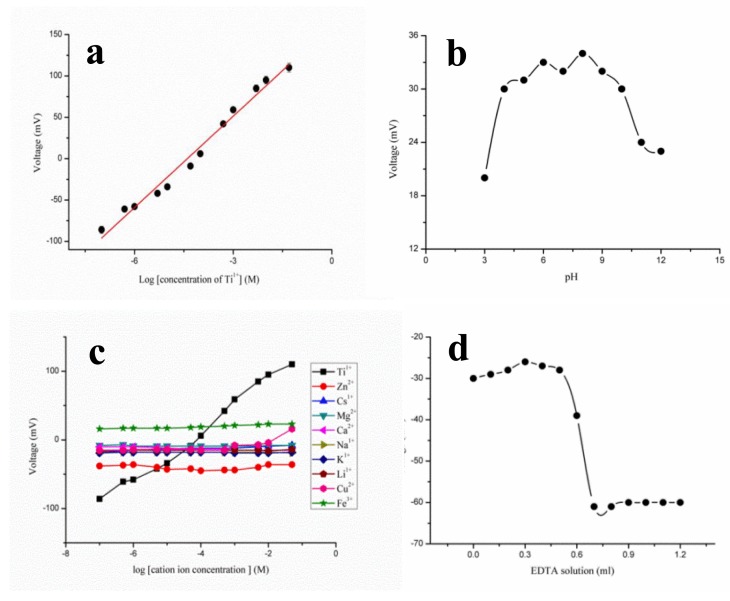
(**a**) Calibration curve for thallium ion sensor in the concentration range from 1 × 10^−7^ to 5 × 10^−2^ M; (**b**) pH effect on the thallium ion sensor from pH 3–12; (**c**) interference curve of thallium ion sensor, and (**d**) potentiometric titration curve of thallium ion sensor for the 18 ml volume of 2 × 10^−3^ M thallium nitrate solution with 5 × 10^−2^ M EDTA solution [84].

ZnO nanorods were synthesized by a hydrothermal method on a gold coated glass substrate, and they were functionalized with an ionophore polymeric membrane for the detection of thallium (I) ion [84]. The output potential of polymer membrane-coated ZnO nanorod-based ion selective electrode depends on the number of charges of electrolytes present in the testing solution. The polymer coated ZnO nanorods were used with a thallium (I) concentration from 1 × 10^−7^ to 1 × 10^−1^ M, and the observed linear range was from 1 × 10^−7^ to 5 × 10^−2^ M for the currently described sensor electrode. The output potential was low at thallium (I) ion concentrations above 5 × 10^−2^ M, which could be due to the saturation limit of the proposed polymer-coated ZnO nanorod-based ion selective electrode. Figure 7a shows the output signal of the currently described sensor device for the thallium (I) ion concentration range of 1 × 10^−7^ to 5 × 10^−2^ M. The observed sensitivity and regression coefficient were 36.87 ± 1.49 mV/decade and 0.98, respectively. The advantageous features of the currently described polymer-coated ZnO nanorods with a selective thallium (I) ion ionophoredibenzyldiaza-18-crown-6 (DBzDA18C_6_) are a low limit of detection (1 × 10^−7^ M thallium ion concentration) and a rapid response time of less than 5 s. All the obtained results, such as high linearity, low limit of detection, sensitivity, and fast response time of the developed polymer-coated ZnO nanorods, demonstrate the potential application of the currently described sensor device for the monitoring of trace quantities of thallium (I) ion from biological and environmental samples.

Figure 7b shows the effect of pH on the output potential response of the fabricated sensor device. The selected pH range was 3–12. As shown in Figure 7b, the electromotive force of the sensor device for pH 4–10 is similar; however, above pH 10, a lower electrochemical response was observed, which could be attributed to the involvement of the hydroxyl (OH^−1^) ion. Concurrently, a low output signal was obtained for low pH values below 4, which was mainly due to the possible dissolution of ZnO nanorods in the acidic pH as demonstrated in previously published work [85]. The working performance of the sensor device is evaluated by various parameters; however, selectivity is the main element that defines the sensing response that is attributable only to the target analyte. The selectivity of the proposed thallium (I) ion sensor device was examined by conducting a different set of experiments. In the first experiment, the mixed method was followed, and the sensor device was inserted in the detected range of thallium (I) concentration in the presence of one mL of individual interfering compound that was added to the testing solution of the thallium electrolyte. A negligible response was observed from the output signal of the sensor device as shown in Figure 7c. The effect of the volume of the interfering compound on the output signal was also investigated, and no significant change in the output signal was observed. In the second experiment, a separation solution method was followed for the calculation of the selectivity coefficient, and the polymeric membrane-coated ZnO nanorod-based ion selective electrode was tested separately in solutions of thallium electrolyte and interfering substances. The measured selectivity coefficient values were determined to be fairly constant for individual interferences as indicated in Table 1. Finally, it was concluded that the polymeric membrane-coated ZnO nanorod-based thallium (I) ion sensor device is highly selective.

Reproducible responses were observed for five independent sensor devices fabricated under the same set of conditions, and the devices were tested at a 1 × 10^−4^ M concentration of thallium (I) electrolyte. It was observed that the sensor had a highly reproducible response with a relative standard deviation <3%.

The shelf-life of the proposed thallium (I) sensor electrode was monitored for a period of four weeks. The polymeric membrane-coated ZnO nanorod-based thallium (I) ion sensor electrode is highly capable of maintaining the detection range, sensitivity, and repeatability and almost exhibited a Nernst response; however, in the fourth week, the detection range was changed from 1 × 10^−7^ to 1 × 10^−6^ M as shown in Table 2. The decrease in the detection range could be due to the slight loss of polymeric membrane from the surface of the ZnO nanorods with time. The analytical application of the fabricated sensor device was demonstrated using the polymeric membrane-coated ZnO nanorod-based ion selective electrode as an indicator electrode in the potentiometric titration at room temperature. Figure 7d shows the analytical application-based response of the proposed thallium (I) ion sensor device by titrating the 18 ml volume of 2 × 10^−3^ M thallium (I) ion *vs.* 5 × 10^−2^ M EDTA solution. The titration curve shows an acceptable stoichiometric relationship for the determination of thallium (I) ion from an unknown sample.

## 6. Glucose Oxidase Immobilized Metal Oxide Nanostructures for the Detection of Glucose

ZnO nanocombs were synthesized by vapor-phase deposition in bulk with high porosity in three dimensions with a large specific surface area and favorable biocompatibility for large quantities of glucose oxidase [86]. The metal oxide nanostructures exhibiting a similarly suitable microenvironment resulted in the creation of a sensor device with a high sensitivity of 15.33 μA·mM^−1^·cm^−2^ and a significant attraction for the loading of glucose oxidase resulting in a Michaelis-Menten constant (Km) of 2.19 mM. A metal oxide nanostructure of ZnO nanotubes was formed by the chemical etching of ZnO nanorods, and the gold electrode was modified with these nanostructures. Glucose oxidase was immobilized on these nanostructures, and the enhanced response of the device was recorded [87]. The hollow structure of ZnO nanotubes exhibited an excellent response without the use of a mediator. The glucose had a wide linear detection range, low limit of detection (1 μM), a sensitivity of 21.7 μA·mM^−1^·cm^−2^ and Km of 21.7 mM. A single ZnO nanofiber with 195 nm to 350 nm diameters was prepared by electro spinning, and it was used for the fabrication of a sensitive amperometric glucose biosensor with a high sensitivity of 70.2 μA·mM^−1^·cm^−2^ because of the large surface area possessed by the ZnO nanostructures, which further exposes large surfaces for the heavy loading of glucose oxidase. A favorable microenvironment has been provided that maintains the bioactivity of glucose oxidase, and the designed device was highly stable for the temperature range of 20 °C to 85 °C [88]. A carbon functionalized ZnO nanowire-based electrode was fabricated for the biosensing of glucose without the use of a mediator by immobilizing glucose oxidase and horseradish peroxidase, and it was shown to have a high sensitivity and low limit of detection for glucose monitoring [89]. The carbon decorated ZnO nanowires crystalline array exhibited a rapid and direct electrochemical platform for glucose oxidase. The intracellular determination of glucose was performed by immobilizing glucose oxidase from human adipocytes and frog oocytes on hexagonal faces of ZnO nanorods grown in a silver coated glass capillary (0.7 μm in diameter) [90]. ZnO nanowires exhibited 60% ionic bonding characteristics and they were very stable at the physiological pH of 7.3. The intracellular biosensor device exhibited a rapid response time and wide spectrum for the detection of glucose molecules. In addition to other metal oxide nanostructures, cerium oxide (CeO_2_) is also well known due to its high isoelectric point of 9.4. It possesses a high electron transfer rate and good surface coverage, which enables the heavy loading of enzyme and rapid electron transfer between the effective glucose oxidase sites and the electrode itself. CeO_2_ films were produced on the platinum coated glass plates by pulsed laser deposition for the functionalization of glucose oxidase, and the glucose biosensor that was developed based on cerium oxide exhibited a linear response across the glucose concentration range, and the magnitude of Km was determined to be 1.01 mM. This type of biosensor is not cost effective and requires special expertise for its operation. In addition, it has a narrow range of glucose detection and a weak limit of detection [91].

**Figure 8.**
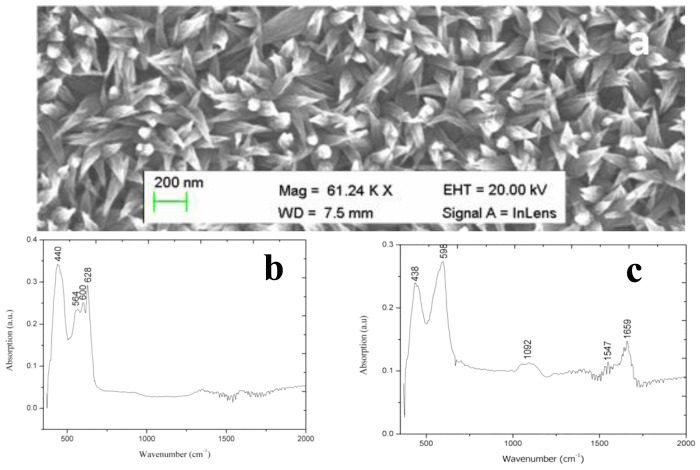
(**a**) SEM images of CuO nanoleaves; (**b**) IRAS spectrum of the pure CuO nanoleaves; and (**c**) IRAS Spectrum of CuO nanoleaves with immobilized glucose oxidase [92].

**Figure 9.**
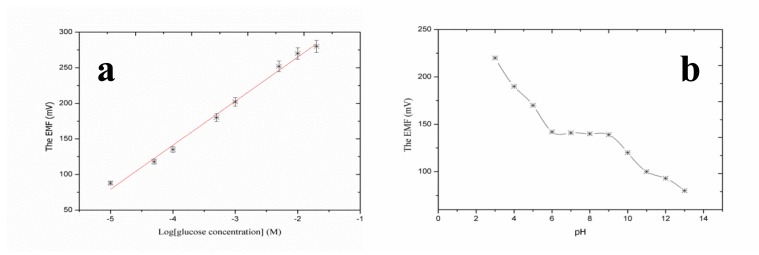
(**a**) Calibration curve of the glucose sensor and (**b**) the effect of pH on the glucose sensor from pH 3 to pH 12 [92].

After their successful synthesis, CuO nanoleaves synthesized using polyethylene glycol as a growth template by a hydrothermal method were used for the chemical sensing of glucose [92]. A representative SEM image of CuO nanoleaves is shown in Figure 8a. The IRAS technique was used for the confirmation of Cu-O bonding in synthesized CuO nanomaterial as shown in Figure 8b. The dominant peaks in the range of 400 cm^−1^ to 600 cm^−1^ are attributed to metal oxide bonds. The observed peaks for 400 to 850 cm^−1^ are assigned to the lattice vibration of Cu-O, O-Cu-O, and Cu-O-Cu [92]. During the measurement of IRAS in this study, different Cu-O peaks were observed at 440, 564, 600, and 628 cm^−1^, which could be indexed to the Cu-O stretching vibration mode [93]. An IRAS study of the glucose oxidase immobilized CuO nanostructures was also performed during which some of these peaks were undetected and some new peaks were detected, which indicates the possible interaction among glucose oxidase molecules and CuO nanostructures. The most characteristics peaks for protein were observed at 1547 cm^−1^and 1659 cm^−1^, which could correspond to amide I and amide II of glucose oxidase as depicted in Figure 8c. The glucose oxidase immobilized CuO nanoleaves were potentially utilized for the chemical sensing of glucose, and the stable output signal was obtained by soaking the CuO nanoleaves-based electrode in the pH 7.3 phosphate buffer solution prior to insertion into the glucose solutions. First, a sensor device was used in the solution of 1.0 × 10^−6^ M glucose, and the observations indicated that the device has the ability to sense glucose at this low concentration, but the output signal was very close to that of the phosphate buffer solution. However, a very strong output signal was observed when the fabricated sensor device was used in the 5.0 × 10^−6^ M glucose solution. Afterward, several solutions of higher concentrations of glucose were prepared in the pH 7.3 phosphate buffer solution and the fabricated sensor device was employed in these concentrations of glucose. A linear range of 1.0 × 10^−5^ to 5.0 × 10^−2^ M was determined for the developed sensor device. Moreover, at concentrations greater than 5.0 × 10^−2^ M glucose, a saturated response was exhibited by the sensor device. The fabricated glucose biosensor exhibited a sensitivity of 61.962.0 mV/decade and a regression coefficient of r^2^ = 0.99. The high sensitivity of the developed sensor device could be attributed to the strong binding of glucose oxidase with CuO nanostructures. The fabricated CuO nanostructures are porous in nature; therefore, they can carry a maximum number of glucose molecules, which results in the high sensitivity of the sensor device as shown in Figure 9a. The low limit of detection of the fabricated device was observed at 5.0 × 10^−5^ M glucose, and a rapid response time of less than 5 s was also demonstrated by the glucose oxidase immobilized CuO nanoleaves.

The sensing mechanism of a glucose oxidase-based glucose biosensor is evidenced by the substrate-catalyzed reaction in the vicinity of enzyme molecules. When the experiment is carried out under those conditions, the immobilized glucose oxidase and glucose molecules interact and consequently, a rapid catalyzed reaction occurs.

Additionally, a fast electron communication occurs on the surface of CuO nanostructures because of the presence of glucose oxidase, which results in the rapid oxidation of glucose molecules. CuO nanostructures have the ability to improve the catalytic performance of glucose oxidase molecules. During the interaction of glucose oxidase and glucose molecules in the reaction vessel, reaction products, including d-gluconolactone and hydrogen peroxide, are produced. The concentration of glucose can be estimated based on these two reaction products or the reduction in the amount of oxygen consumed in the oxidation of glucose molecules. Gluconolactone is an unstable molecule that is rapidly converted into gluconic acid, which then reacts with water and is converted into gluconate and hydronium ions as depicted in the following chemical reaction:

(2) $${\text{H}}_{2}\text{O}+{\text{O}}_{2}+\text{D-glucose GOD}\to \text{D-gluconolactone}+{\text{H}}_{2}{\text{O}}_{2}$$

(3) $$\text{D-gluconolactone spontaneously}\to {\text{gluconate}}^{-}+{\text{H}}_{3}{\text{O}}^{+}$$

Because of the production of hydronium and gluconate ions in the reaction vessel and in the solution, these charges move over the surface of semiconducting nanomaterial resulting in strong electrical signals that cause electromotive generation during the reaction of glucose oxidase and glucose molecules.

The response of different sensors is known as reproducibility and it defines the similar trends of sensor devices. For this experiment, nine CuO nanoleaves-based sensor devices were fabricated by immobilizing glucose oxidase. The currently described sensor devices were tested in 1.0 × 10^−4^ M glucose at standard conditions of temperature and pressure. The response of these sensor devices was reproducible, and it can be observed that response between sensor devices varied by a standard deviation of less than 5%. The repeatability measures the response of sensor devices used more than once, and for this experiment the same sensor device was used for four consecutive days. The proposed sensor retains its detection range as well as sensitivity, and the results of this experiment are summarized in Table 1. Selectivity is an important parameter for the evaluation of the performance of sensor devices and it measures the sensor sensing capability for the target analyte in the presence of other competing species. The selectivity of a glucose sensor is investigated by two methods: enzyme-substrate reaction and the selective response for the glucose molecules. The biological reactions catalyzed by enzymes are highly selective; therefore, glucose oxidase only oxidizes glucose molecules even in the presence of bioactive compounds, including the sugars D-galactose, D-mannose, and D-ribose. However, some reducing species, such as ascorbic acid, L-cysteine, and uric acid, are investigated as common interferences for glucose detection using an amperometric technique. The physiological concentration of ascorbic acid is approximately 1.0 × 10^−4^ M in the human serum [94], and we evaluated the response of the current glucose sensor in this concentration range. Each interfering compound exhibited a negligible response. This investigation has proved that the sensor device is highly selective and functions well under the physiological concentrations of ascorbic acid, uric acid, and L-cysteine. The lifetime of the proposed glucose sensor was monitored for three weeks, and the developed sensor device maintains its sensitivity and detection range. For potentiometric sensor devices, it is highly important to investigate the effect of pH on the output signal of fabricated sensor devices. CuO is an amphoteric compound; thus it is not stable in highly acidic and alkaline media. The pH effect was studied for the 1.0 × 10^−4^ M concentration of glucose, and the pH of the testing solution was controlled by adding 0.01 M hydrochloric acid and 0.01 M sodium hydroxide. The pH range selected for this experiment was 3 to 13 as shown in Figure 9b. It can be observed in Figure 9b that the sensor device is highly sensitive to acidic pH. The high output signal could be due to the possible interaction between the hydronium ions and the immobilized glucose oxidase enzyme. However, for pH 6 to pH 9, the output potential was observed fairly consistently, and at this pH range the response could be due to the oxidation of glucose molecules. Thus, pH 7.3 was selected for all experiments. Above pH 9, a decreased output signal was observed, which could result from the instability of CuO at higher alkaline pH values, and the dissolution of CuO is possible at higher alkaline pH values.

## 7. Cholesterol Oxidase Functionalized Metal Oxide Nanostructures for the Detection of Cholesterol

**Figure 10.**
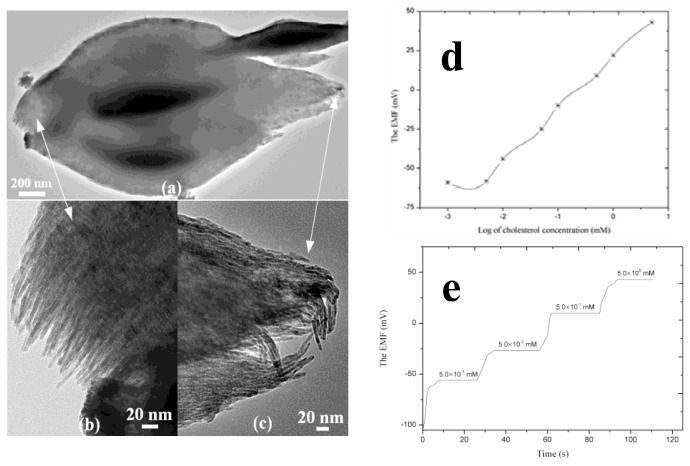
(**a**–**c**) TEM images of nanowire bundles; (**d**) calibration curve of cholesterol biosensor with detection limit; and (**e**) response time of the cholesterol biosensor in the different cholesterol concentrations [98].

Different metal oxide nanostructures have been employed for the development of cholesterol biosensors by immobilizing the cholesterol oxidase on the surface of metal oxide nanostructures. Among these metal oxides, ZnO is widely used for the immobilization of cholesterol oxidase due to the large difference of isoelectric points. A sol-gel method was used to fabricate a cholesterol biosensor with a wide linear range of 5.0 mg·dL^−1^ to 400 mg·dL^−1^, small Km (0.98 mg·dL^−1^) and high sensitivity of 59 nAmg^−1^·dL·cm^−2^ [95]. The fabricated cholesterol biosensors can quantify the cholesterol concentration in serum/blood samples. An electrode of platinum added to fullerene-like ZnO hybrid nanospheres (50–200 nm) has been developed using the electrode position of the nanospheres on the surface of a glassy carbon electrode [96]. This electrode exhibited an enhanced sensitivity of 1.886 mA·mM^−1^·cm^−2^ in producing high enzymatic activity. The combination of ZnO and platinum nanoparticles reduces the applied potential in the amperometric estimation of cholesterol with improved reduction of interferences. Enhanced sensitivity and a low limit of detection have been observed for the cholesterol biosensor fabricated with the gold electrode modified with ZnO nanostructures [97]. Recently, a CuO bundle of nanowires was synthesized by a hydrothermal method and it was used for the chemical sensing of cholesterol [98]. A bright field TEM image of bundle of nanowires is shown in Figure 10a–c. Cholesterol oxidase was physically adsorbed on the surface of the CuO bundle of nanowires. The electromotive force response of cholesterol oxidase immobilized CuO nanostructures was measured by a potentiometric technique in a phosphate buffer solution of pH 7.30 at room temperature. Figure 10d shows the electrochemical response of the presented sensor device for the cholesterol concentrations, and the observed results indicate the linear range for the proposed device was 5.00 × 10^−3^ to 10^0^ mM, and the detection limit was determined to be 1.00 × 10^−3^ mM. This cholesterol biosensor exhibited a sensitivity of 33.88 ± 0.96 mV/decade and a regression coefficient of 0.99. A rapid response time of less than 10 s was exhibited by the sensor device as shown in Figure 10e. The excellent performance of the potentiometric cholesterol biosensor could result from the heavy loading of cholesterol oxidase on the sharp edged surface of the CuO bundle of nanowires. These sharp edges exposed a favorable microenvironment for the improved catalytic activity of the enzyme. The sensing mechanism of the fabricated sensor device can be explained by two different possibilities: (1) cholesterol oxidase is very selective towards the oxidation of cholesterol molecules and (2) cholesterol oxidase has a favorable catalytic efficiency for the enhancement of chemical reactions via the direct interaction of cholesterol molecules with the immobilized cholesterol oxidase on the CuO nanomaterial. The flowing reaction products are produced during the catalytic oxidation of cholesterol molecules in the reaction vessel [99]:

(4) $$\text{Cholesterol}+{\text{O}}_{2}\to 5–3‐\text{ketosteroid}+{\text{H}}_{2}{\text{O}}_{2}$$

The 5–3-ketosteroid is the intermediate and it has short lifetime during the reaction. It forms spontaneously via the isomerization of the trans-double bond at the 5–6 position of the steroid ring by an intramolecular change of the proton from the 4–6 β position resulting in the formation of a stable product, 4–3-ketosteroid, as shown in the chemical Equation (5):

(5) 5–3‐ketosteroid→isomerization4–3‐ketosteroid

The generation of output potential for the fabricated sensor device could result from the afore-mentioned reaction mechanism, which creates a charged environment around the working electrode, and the resulting potential difference can be measured using a pH meter. It has been shown that CuO itself exhibits catalytic properties that can improve the efficiency of cholesterol oxidase and create a rapid and direct electron transfer rate between the active sites of cholesterol oxidase and its own surface. Moreover, the fabricated cholesterol biosensor using a bundle of CuO nanowires was found to be highly reproducible, repeatable, stable and selective (data not shown).

## 8. Polymer Membrane-Coated CuO Nanoflowers for the Selective Detection of Cadmium Ion

**Figure 11.**
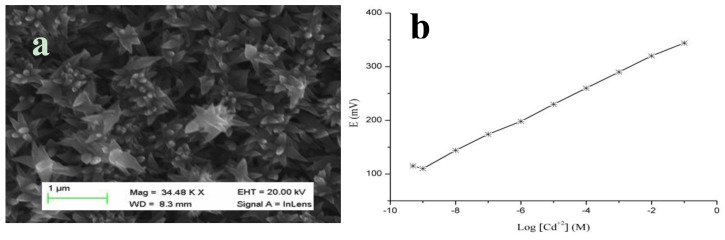
(**a**) SEM image of CuO nanoflowers and (**b**) calibration curve of cadmium ion sensor-based CuO flowers within the limit of detection [100].

CuO nanoflowers were fabricated on a gold-coated glass substrate by a hydrothermal method [100], and a representative SEM image is shown in Figure 11a. CuO is well known for its high electrochemical response; thus, it is frequently used in the development of electrochemical batteries. In the currently described study, it functions as an anode material. To maintain these valuable properties of CuO, we grew flower-like nanostructures using urea by a chemical growth method that was also utilized in the fabrication of a cadmium ion sensor. The polymeric membrane containing tetrathia-12-crown-4 as a selective ionophore for the chemical sensing of cadmium ion was coated on the surface of the flower-like morphology of CuO [101]. Tetrathia-12-crown-4 exhibits soft base like properties; therefore, it has a potential ability to form a complex with soft metal ions, such as cadmium, which has been demonstrated in a published study [101].

CuO nanostructures are associated with certain unique properties, including fast electron communication, high surface-to-volume ratio, and well-established transducing features for the production of strong electrical signals. The cadmium ion sensor described here has a wide detection range of 1.0 × 10^−9^ M to 1.0 × 10^−1^ M with a Nernstian slope of 29.3 ± 0.3 mV/decade. The regression coefficient value of 0.99 revealed the excellent analytical device properties for the selective sensing of cadmium ion. Figure 11b demonstrates the wide range of detection of cadmium ions by the proposed sensor device with an acceptable Nernstian response. The wide range of sensing of cadmium ion by coating the tetrathia-12-crown-4 ionophore membrane on CuO nanostructures could be due to a high electro-catalytic property, high surface-to-volume ratio, potential electron transport and rapid complex formation between the cadmium ion and selective ionophore. These factors account for the excellent sensitivity, stability and a rapid response time of the fabricated sensor device. A rapid response time of less than 10 s was exhibited by the developed cadmium ion sensor using the polymer-coated CuO nanoflowers.

## 9. Polymer-Coated Honeycomb-Like Morphology of Nickel Oxide for the Chemical Sensing of Zinc Ion

A nickel oxide honeycomb-like morphology was fabricated on the nickel foam by a hydrothermal method, and nanostructures were potentially used for the development of a zinc ion sensor [102]. The polymer membrane of 12-crown-4 was coated on the NiO nanostructures, and selectively properties were used for the sensing of the zinc ion. The electrochemical response of the polymer membrane-coated honeycomb-like nanostructures was measured for the zinc ion concentration range of 0.0005 mM to 100 mM. The fabricated sensor has the ability to sense the 0.0005 mM concentration of the zinc ion, but it deviates from the linear range of the sensor device. The linear range response for the fabricated device was found to be 0.001 mM to 100 mM with a sensitivity of 36 mV/decade and a regression coefficient of 0.99. This is attributed to the large surface provided by the honeycomb-like nanostructures for the coating of the polymeric membrane. Additionally, the NiO nanostructures reduce the diffusion path in the solid phase, which could be responsible for the higher sensitivity compared to that of the Nernstian 29 mV/decade. The lower limit of detection was determined to be 0.0005 mM and a rapid response time of less than 10 s was observed for honeycomb nanostructures coated with a polymeric membrane consisting of an ionophore that is selective for zinc ion.

The optical, electrical and magnetic characteristics of metal oxide nanostructures have been explored and published with improved performance via the addition of nanoparticles of conducting or semiconducting nanomaterials, including carbon nanotubes, graphene, gold and silver. Additionally, quantum dots of different semiconductors have advantageous features for the enhanced sensitivity and low detection limit properties of biosensors [103–107].

## 10. Conclusions

The functionalization of biosensitive materials by maintaining the stability of material has recently attracted increased attention among the research community. Different strategies have been proposed to achieve the desired goals. The goal of the concept of molecular imprinting is to optimize the selectivity of well-known artificial materials, polymer-thin film coatings, and self-assembled monolayers that have specific functions [45]. However, nanostructures are well-known for their sensitive surface and selectivity, and they have the ability to maintain the configuration of the device. Different metal oxide nanostructures have been used for molecular and ion recognition. However, there is still ample opportunity for the development of sensitive and reliable devices using nanostructures for practical applications.

Metal oxide nanostructures-based sensor devices have provided a straightforward and novel route for new functions having a wide range of applications in clinical and non-clinical diagnosis. Different metal oxide nanostructures have shown well known impact in different fields including enzyme electrodes to genoelectronics. The attractive properties of various metal oxide nanostructures give a logical clue about the future interdisciplinary fields is most likely move to a novel generation of electrochemical biosensors. The unique properties of metal oxide nanostructures could lead to the development of new biosensors with improved signal amplification and coding sequence that could be used with biosensitive materials and effective electrical communication for redox biomaterials, such as enzymes that can increase their future diagnostic demands. The favorable and unique characteristics of metal oxide nanostructures exhibit significant interfacial biological recognition events through electronic signal transduction for the development of novel bioelectronics analytical tools with improved new functions. The tightly regulated synthesis of metal oxide nanostructures has a contributing role in the design of novel biosensors. Significant effort has been expended to expose the development and future problems associated with metal oxide nanostructures for the engineering of biosensing devices and the exploration of novel systematic strategies for bio-affinity configuration and effective electrical communication. The wide range of newly prepared metal oxide nanostructures increases the diversity of the biosensor field. Different synthetic routes for the preparation of one-dimensional metal oxide nanostructures are likely to open the door for novel bioelectronics sensing applications. The relative simplicity for the functionalization of the surface of metal oxide nanostructures with specific groups that can be attached to the target biomolecules, even in the presence of impurities, results in the enhanced electronic properties of electroactive materials toward achieving improved charge transfer, which can be a suitable methodology for the development of sensitive biosensor devices. The fabrication of metal oxide nanostructures in diverse morphologies with similar nano dimensions could provide a favorable microenvironment for the immobilization of biosensitive materials that can further amplify the electrical signals. The synthesis of metal oxide nanostructures in different morphologies can be used for several applications, such as sensor arrays for the engineering of functional integrated nanodevices. The attractive surface of metal oxide nanostructures could be useful for the real-time monitoring of biomolecules with multi-sensory capabilities. The testing of novel methods for the development of new biosensors for use in health care settings is in high demand. The development of biosensors that can bind with various biomolecules on the closely packed one-dimensional nanostructure of metal oxides is also needed. .

## Figures and Tables

**Table 1.** The logarithm of the selectivity coefficient of the thallium ion sensor for different interferents in 1 × 10^−4^ M [84].

**Interference (B)**	$-\log K_{\left(\text{Tl}\;B\right)}^{\text{pot}}$
K^1+^	4.65
Ca^2+^	4.63
Na^1+^	4.66
Mg^2+^	4.40
Li^1+^	4.55
Cu^2+^	4.11
Cs^1+^	4.33
Fe^3+^	3.50

**Table 2.** Representing the durability of thallium (I) ion sensor [84].

**Number of days**	**Slope (mV/decade)**	**Linear range ( M )**
1 day	36.87 ± 1.49	1 × 10^−7^–5 × 10^−2^
1 week	37.10 ± 2.20	1 × 10^−7^–5 × 10^−2^
2 weeks	36.62 ± 2.44	1 × 10^−7^–5 × 10^−2^
3 weeks	36.69 ± 2.56	1 × 10^−7^–5 × 10^−2^
4 weeks	35.53 ± 1.12	1 × 10^−6^–5 × 10^−2^
